# Learning a locomotor task: with or without errors?

**DOI:** 10.1186/1743-0003-11-25

**Published:** 2014-03-04

**Authors:** Laura Marchal–Crespo, Jasmin Schneider, Lukas Jaeger, Robert Riener

**Affiliations:** 1 Sensory-Motor Systems Lab, Institute of Robotics and Intelligent Systems IRIS, ETH Zurich, Zurich, Switzerland; 2 Institute of Human Movement Sciences and Sport, ETH Zurich, Zurich, Switzerland; 3 Clinic for Neuroradiology, University Hospital Zurich, Zurich, Switzerland; 4 Medical Faculty, Balgrist University Hospital, University of Zurich, Zurich, Switzerland

**Keywords:** Error amplification, Noise disturbance, Haptic guidance, Motor learning, Locomotor rehabilitation, Simple task, MRI-compatible robot

## Abstract

**Background:**

Robotic haptic guidance is the most commonly used robotic training strategy to reduce performance errors while training. However, research on motor learning has emphasized that errors are a fundamental neural signal that drive motor adaptation. Thus, researchers have proposed robotic therapy algorithms that amplify movement errors rather than decrease them. However, to date, no study has analyzed with precision which training strategy is the most appropriate to learn an especially simple task.

**Methods:**

In this study, the impact of robotic training strategies that amplify or reduce errors on muscle activation and motor learning of a simple locomotor task was investigated in twenty two healthy subjects. The experiment was conducted with the MAgnetic Resonance COmpatible Stepper (MARCOS) a special robotic device developed for investigations in the MR scanner. The robot moved the dominant leg passively and the subject was requested to actively synchronize the non-dominant leg to achieve an alternating stepping-like movement. Learning with four different training strategies that reduce or amplify errors was evaluated: (i) Haptic guidance: errors were eliminated by passively moving the limbs, (ii) No guidance: no robot disturbances were presented, (iii) Error amplification: existing errors were amplified with repulsive forces, (iv) Noise disturbance: errors were evoked intentionally with a randomly-varying force disturbance on top of the no guidance strategy. Additionally, the activation of four lower limb muscles was measured by the means of surface electromyography (EMG).

**Results:**

Strategies that reduce or do not amplify errors limit muscle activation during training and result in poor learning gains. Adding random disturbing forces during training seems to increase attention, and therefore improve motor learning. Error amplification seems to be the most suitable strategy for initially less skilled subjects, perhaps because subjects could better detect their errors and correct them.

**Conclusions:**

Error strategies have a great potential to evoke higher muscle activation and provoke better motor learning of simple tasks. Neuroimaging evaluation of brain regions involved in learning can provide valuable information on observed behavioral outcomes related to learning processes. The impacts of these strategies on neurological patients need further investigations.

## Background

Recent work in robotic assistance has focused on developing sophisticated robotic mechanisms in order to support movement training of more complex movements such as walking [1-4]. Although robotic locomotor training has been proposed as a promising technique to improve rehabilitation in patients with severe gait impairments, the functional gains obtained after robotic training are still limited [5,6]. During robotic training, the patients are assisted with body weight support as needed and provided with haptic guidance from a gait orthosis to move their legs into a correct gait kinematic pattern. Robotic haptic guidance is a motor-training strategy in which a machine physically guides the subject’s limbs during movement training. It is believed that haptic guidance could provide the central nervous system with additional proprioceptive and somatosensory cues to enhance movement planning, as well as enable to attempt more advanced strategies of movement that are dangerous to practice, such as learning to walk after a neurologic injury [4].

Although robotic guidance is often used in motor training, there is currently little evidence that robotic guidance is more beneficial for human motor learning than unassisted practice. In fact, a long-standing hypothesis in motor learning research is the Guidance Hypothesis which states that physically guiding a movement impairs motor learning because the user learns to rely on the guidance and fails to learn the motor commands required to perform the desired task [7]. Patient’s effort during physical training is thought to be an important factor in order to provoke motor plasticity [8], thereby robotic devices could potentially decrease recovery if they encourage a decrease in effort, energy consumption, or attention during training [9]. A recent randomized study showed that patients with incomplete SCI only obtained marginal improvements in overground walking speed after 12 weeks of robotic-assisted training [6]. A possible explanation why conventional therapist-assisted training seems to outperform robotic rehabilitation is the inability of the controllers to adapt to the patients special needs.

Assisting robotic therapy strategies reduce movement errors, i.e. they help the subject to do the task better. However, research on motor learning has emphasized that errors are fundamental signals that drive motor adaptation [10-12]. Previous studies have shown that healthy subjects can adapt to a force perturbation during stepping [13,14]. Patients with incomplete SCI seem to preserve the ability to adapt when experiencing a force perturbation during walking [15]. Since errors drive motor adaptation [13], error-amplification training may induce more robust aftereffects as compared to assistive locomotor training [16].

There has been a progression in the development of control strategies that amplify movement errors rather than decrease them (i.e. challenge-based robotic controllers) [17]. In patients with chronic stroke, amplifying errors during reaching with a robotic force field resulted in straighter movements when the force field was removed [11]. Similarly, increasing limb phasing error in post-stroke participants’ gait through a split-belt treadmill induced a long term increase in walking symmetry [12]. Training a reaching task with error amplification was more beneficial for less impaired stroke patients, whereas more impaired patients benefited more from haptic guidance [18]. This result is consistent with [19], where training with amplified errors produced greater learning to play a pinball-like game than training with haptic guidance in higher-skilled participants, while for the less-skilled participants, training with haptic guidance was more beneficial.

An extended approach to error amplification is noise disturbance: randomly-varying feedforward forces that disturb subjects’ movements during training. In a recent study [20], training with noise disturbance resulted in better tracking than unassisted training and than training with a more conventional error-amplification strategy (repulsive forces proportional to tracking errors). The question of the most effective control algorithms, and their relative benefits compared to unassisted practice still remains unanswered [21]. Matching the robotic training strategy to the trainee’s skill level may provide the greatest opportunity for learning.

Haptic guidance seemed to be particularly helpful for less skilled subjects, while error amplification was found to be more beneficial for more skilled participants [18,19]. This is in line with the challenge point theory, that states that optimal learning is achieved when the difficulty of the task is appropriate for the individual participants level of expertise. Thus, providing an easy task to a proficient participant would not be predicted to improve learning, since little new information is delivered and new skills are not mastered. Previous error-amplification experiments have focused mainly on complex tasks (e.g. reaching a target and walking on a treadmill). Complex tasks have been defined as tasks that cannot be mastered in a single practice session and have several degrees of freedom [22]. However, based on the challenge point theory, error-amplification strategies may be more suitable to enhance learning of simple tasks (i.e. tasks with only one degree of freedom, that can be mastered in a single practice session, and that seem to be artificial [22]).

Motor learning, and exercises themselves, are incredibly diverse. Studying the training strategy that optimizes motor learning might help to get an insight in patients’ gait rehabilitation [23]. Cortical changes have been shown to occur only with learning of new skills and not just with repetitive use [8,24], suggesting that motor learning mechanisms are operative and critical during brain plastic recovery. Understanding the underlying mechanisms of motor learning during robotic locomotor training is crucial to improve the efficacy of robotic training in patients [25]. We seek to tailor the control algorithm to the patient-specific recovery stage to improve robotic training. In this study, the impact of four different training strategies on muscle activation and on learning a especially simple task was tested with twenty two healthy subjects. The MAgnetic Resonance COmpatible Stepper (MARCOS), a one-degree-of-freedom robot, was used to conduct the experiment under different training modes: with haptic guidance, without guidance, with error amplification (i.e. repulsive forces proportional to errors), and in noise-force disturbance mode (i.e with a randomly varying force disturbance added to the no haptic guidance mode).

We hypothesize that the tracking error, as well as the muscle activation, are higher when training with strategies that amplify errors compared to the passive and the active modes without error disturbance. Additionally, we expect better motor learning and aftereffects after training with the challenged-based strategies.

## Methods

### MARCOS

**Figure 1:**
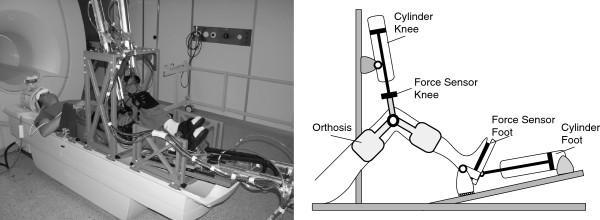
**The fMRI compatible robotic stepping actuator MARCOS.** Left: MARCOS in the 1.5T MR scanner. Right: Sketch of the MARCOS system (only 1 leg depicted for clarity). MARCOS is actuated by two pneumatic cylinders per leg. The reaction forces between the subject and the robot are measured through force sensors located in the knee orthosis attachment and the foot plate. The position of each cylinder piston is measured redundantly by optical encoders [26].

MARCOS (Figure 1) is a one-degree-of-freedom pneumatic robotic device that enables the assessment of brain activation using functional magnetic resonance imaging (fMRI) during gait-like stepping movements. MARCOS is actuated by two pneumatic cylinders per leg (Figure 1). The pneumatic cylinder attached to a knee orthosis can move the knee up and down, while the feet of the subject (placed in a shoe and fixed with Velcro fasteners) slides on a linear guide. A second pneumatic cylinder is attached to the shoe on the linear guide and allows the control of a force at the foot sole that simulates the ground reaction forces. Proportional way valves (MPYE, Festo, Esslingen am Neckar, Germany) control the air flow to the knee cylinders. The cylinders attached to the shoes are controlled with pressure control valves (VPPM, Festo, Esslingen am Neckar, Germany). A proportional way valve in series with this proportional pressure valve distributes the pressure to both chambers.

The arrangement of the knee and foot actuation allows predefined flexion and extension movements of each leg individually in the sagittal plane that resemble on-the-spot stepping, including rotation about the hip, knee and ankle joints. The human-robot reaction forces are measured through force sensors located in the knee-orthosis attachments and the foot plates. The position of each cylinder piston is measured redundantly by optical encoders with a ceramic scale and a foil potentiometer. For more detailed information about the robot design the reader is referred to [27].

### EMG device

Surface EMG was measured using a wireless TeleMyo 2400T Direct Transmission System, the MyoResearch XP Master Edition software and dual silver/silver chloride (Ag/AgCl) snap electrodes (all from Noraxon, Arizona, USA). Data was recorded at 1500 Hz.

Four muscles on each leg were recorded: rectus femoris (RF), biceps femoris (BF), gastrocnemius medialis (GM) and extensor hallucis longus (EHL). The EHL was chosen as substitution for the tibialis anterior (TA), because the TA was fully covered by the knee-orthosis and therefore could not be reliably measured. The EHL was located 1–2 cm lateral from the tibia just distal from the bulge of the TA. The BF and the GM were both located according to the SENIAM guidelines [28]. As the orthosis was covering the predefined location, the RF electrode position recommended by SENIAM had to be changed from 50% to 40% of the line from the anterior superior iliac spine to the superior part of the patella. The skin was shaved when covered with hair, and cleaned with alcohol before placing the electrodes in order to improve the electrode-skin contact.

### Training modes

The experiment consisted of actively synchronizing the non-dominant leg in amplitude and frequency with the dominant leg, passively moved by the robot, to achieve an alternating stepping-like movement. The synchronization was practised under four different training conditions (i.e. modes): i) Haptic Guidance (HG), ii) No Guidance (NG), iii) Error amplification (Ea), and iv) Noise force disturbance (Noise). In haptic-guidance mode, MARCOS guided the gait pattern, while the subject remained passive. In no-guidance mode, the subject was in charge of the movement generation, and the robot followed the subject movements. In error-amplification mode, MARCOS amplified the tracking error created by the subject, adding a force on the knee proportional to the tracking error. In noise-force-disturbance mode, a random force generated by the knee cylinder was superimposed to the active movement in order to disturb the movement generated by the subject.

#### Haptic-guidance mode

The haptic guidance mode combines a feedback position controller with an iterative learning controller (ILC). The position controller enforces the desired knee trajectory through the length of the knee cylinder, controlling the opening of the proportional flow valves. The actuation variable from the position controller is proportional (*P*_*pos*_) to the difference between the desired knee position (*y*_*des*_) and the measured position (*y*_*meas*_):

(1) $$u_{\text{pos}}=P_{\text{pos}}\left(y_{\text{des}}-y_{\text{meas}}\right)$$

The ILC exploits the particular characteristics of the gait cyclic movement to learn how to periodically improve the overall control performance [29]. It calculates a feedforward control signal (*u*_*k*_(*t*)) for the current step *k* out of the error trajectory of the previous steps, in a similar fashion as in [30]:

(2) $$u_{k}(t)=g\cdot e_{k-1}(t+\mathrm{\Delta t})+f\cdot u_{k-1}(t)$$

Thus, the control signal from the ILC at each discrete time *t*, is proportional to the tracking error created in the previous cycle *e*_*k*−1_, and the control signal in the previous cycle *u*_*k*−1_(*t*), at the same discrete time. The proportional gain *g* is the learning gain, and *f* is the robot forgetting factor. We introduced a time shift *Δt* in the tracking error *e*_*k*−1_ to compensate for the delay in the system, due mainly to the long air tubing. The reader is referred to [27] for a completed description of the haptic guidance mode.

#### No-guidance mode

In no-guidance mode the robot follows the subject self-selected movements in such a way that the interaction forces between human and robot are minimized. Thus, the robot is compliant and the subject can move without feeling the robot.

The control strategy for the NG mode is a closed-loop force controller with control gain *P*_1_. The user should not feel the weight of the orthosis *W*, and thus the measured force at each knee (*F*_*meas*_) is controlled to counteract the orthosis weight (*W* = 0.8 kg). The term *P*_2_*xF*_*meas*_ compensates for the dependency of pressure build-up on chamber volume, resulting in a simplified version of the control strategy suggested in [31]. A quadratic term in the force *P*_3_(*W*−*F*_*meas*_)^2^ was added to increase the control output at larger forces:

(3) $$\;u_{\text{knee}}=\left(P_{1}+P_{2}x\right)\left(W-F_{\text{meas}}+P_{3}{\left(W-F_{\text{meas}}\right)}^{2}\right)$$

#### Error-amplification mode

During the error amplification mode, MARCOS amplifies the errors generated when trying to follow a desired knee movement. The actuation variable is proportional to the difference between the desired knee position (*y*_*des*_) and the measured actual position (*y*_*meas*_), similarly to the position controller in the passive mode. However, the proportional gain is negative (*K*_*amp*_ = –2 N/m) in the error-amplification mode. Thereby, the force generated by the knee cylinder is smaller as smaller is the error and increases with the tracking error. The error-amplification controller works on top of a closed-loop force controller by adding the error-amplification control variable to the control variable as follows:

(4) $$\begin{array}{ll} u_{\text{knee}} & =\left(P_{1}+P_{2}x\right)\left(W-F_{\text{meas}}+\mathrm{..}\right)\\ & \;\left(+\;P_{3}{\left(W-F_{\text{meas}}\right)}^{2}\right)+K_{\text{amp}}\left(y_{\text{des}}-y_{\text{meas}}\right) \end{array}$$

We saturated the magnitude of the error-amplification force to guarantee the subjects’ safety and limit the task difficulty.

#### Noise-disturbance mode

A controller that applies random perturbating forces on the knee was designed to test the effect that randomly evoked errors had on motor learning. The knee cylinder applies the disturbance as a random magnitude force every 0.5 seconds that last for 0.1 seconds. The force magnitude is randomly generated by a Band-Limited White Noise block in Simulink, and ranges between ± 100 N. Similarly to the error-amplification mode, the noise disturbance works on top of the closed-loop force controller described above.

### Study protocol

**Figure 2:**
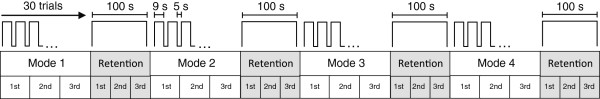
Study protocol.

The study was approved by the local ethical committee (Kantonale Ethikkommission Zürich, Application Number: EK-856) and conducted in compliance with the Declaration of Helsinki. Twenty two healthy subjects (11 male), 23.0 ± 2.0 years old, gave written informed consent to participate in the study. The participant in Figure 1 consented to the publication of his image. All subjects were right footed (Waterloo Handedness Questionnaire [32]).Subjects were instructed to relax the dominant leg while it was moved by the robot, and to synchronize the non-dominant leg with the passive leg to achieve a gait-like alternating movement with same amplitude (0.16 m) and frequency (0.5 Hz). A within-subject cross-over design was used to test the effects of training with the four different training modes (Figure 2). Each training session consisted of 30 trials of 9 s of movement followed by 5 s rest. After each training session, subjects performed a 100 s of sustained movement in no-guidance mode to test retention. All subjects started training with the haptic-guidance mode to help them to understand the task to be performed. During the haptic-guidance condition, subjects were instructed to relax and keep both legs passive. The active modes were tested in randomly order (Figure 2).

### Data processing and statistical analysis

The mean absolute tracking error was calculated as the mean absolute difference between the measured and desired knee positions. The first two seconds after the movement initiation were removed to avoid the negative effect that subjects’ individual reaction times may have on the tracking error. Due to technical problems, the knee position of one subject was not recorded during NG and Noise, and it was not recorded at all in a second subject. The EMG signals were rectified, smoothed with a moving average filter (window length 50 ms) and normalized to the maximum activation of the corresponding muscle over all runs. Data was processed with Matlab.Normal distribution of the data was checked visually using Q-Q plots. The tracking error and muscle activation during training was compared between modes to assess if some modes were more challenging than others using a repeated measure of variance (ANOVA) with Bonferroni correction. In order to analyze adaptation during training, data from each mode were divided into thirds (Figure 2). The mean tracking error, as well as the mean muscle activation, were calculated for each third separately and adaptation was analyzed comparing data between the first and last third for each condition with a paired t-test.

In order to assess learning effects after training with a certain mode, the error created during the retention session after training with the active modes were compared with retention after the HG mode. Not only the HG mode allowed subjects to understand the task, but also the retention session afterwards served as the experiment’s baseline. Thus, the error created during all retention sessions were compared to evaluate learning and to check for condition dependent learning differences with a repeated measure of variance (ANOVA) with Bonferroni correction. We analyzed if training with the challenge-based modes (i.e. Ea and Noise) created aftereffects once training was finished by comparing the muscle activation during retention after Ea and Noise to the activation after NG and HG with a paired t-test.

The analysis was also conducted separately for initially skilled and non-skilled subjects. Subjects were divided into two groups depending on the mean tracking error created during retention after the HG mode - better half to skilled, worse half to non-skilled. The significance level was set at *α*=0.05. If not mentioned otherwise, the estimated values of the repeated measure ANOVA and the standard deviation are stated.

## Results

### Performance during training with different modes

**Figure 3:**
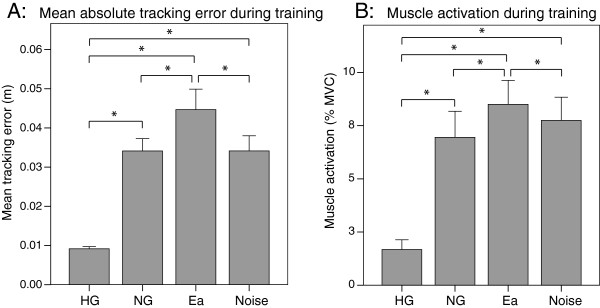
**Performance during training.A:** Mean absolute tracking error during the different training modes: Haptic guidance, No guidance, Error amplification and Noise disturbance. **B:** Percentage of muscle activation during the different training modes. Error bars in all plots show +/- 1 SD. *p < 0.05.

The mean tracking error of the active leg during the different conditions was significantly different between all conditions (Figure 3A, HG vs. NG: *p*<0.001, HG vs. Ea: *p*<0.001, HG vs. Noise: *p*<0.001, NG vs. Ea: *p*<0.001, Ea vs. Noise: *p*<0.001) except between the NG and Noise modes (*p*=1).

Considering the average of the four muscles of the active leg, the HG mode showed a significantly smaller muscle activation than the active modes (Figure 3B, HG vs. NG: *p*<0.001, HG vs. Ea: *p*<0.001, HG vs. Noise: *p*<0.001). Additionally, the muscle activation during Ea was significantly higher than during NG (*p*<0.001), and showed a tendency of being greater than during Noise (*p*=0.065). The Noise mode also showed a tendency of greater activation than the NG mode (*p*=0.203).

Interestingly, the same pattern of muscle activation was found in the passive leg even if subjects were informed to relax the dominant leg. In particular, the HG mode showed a significantly smaller muscle activation than the other modes (HG vs. NG: *p*<0.001, HG vs. Ea: *p*<0.001, HG vs. Noise: *p*=0.004). The muscle activation during Ea was significantly higher than during NG (*p*=0.017) and Noise (*p*=0.005). The NG and Noise conditions did not present significant differences. Although the activation pattern seemed to be similar between the two legs, subjects were able to adjust their right and left leg’s muscle activation separately. The comparison of the same muscle between legs (all conditions included) showed that there was a significantly greater activation in the non-dominant leg muscles (RF: *p*=0.014, BF: *p*=0.016 and GM: *p*=0.002). Only between the right and left EHL there were no significant differences.

### Effect of initial skill level on motor learning

**Figure 4:**
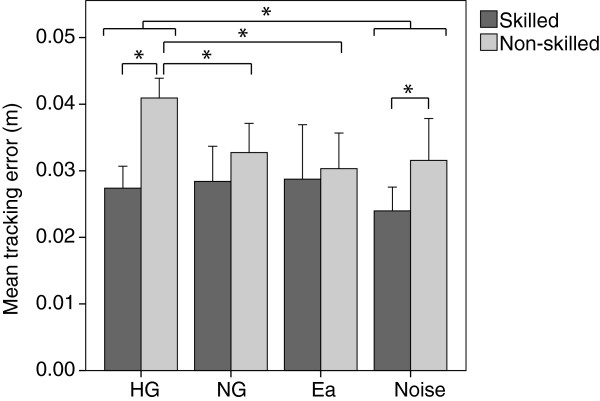
**Mean absolute tracking error for initially skilled and non-skilled subjects during retention after the different training modes: Haptic guidance, No guidance, Error amplification and Noise disturbance.** Error bars in all plots show +/- 1 SD. * *p*<0.05.

Learned occurred only after training with Noise. The tracking error was only significantly reduced from baseline (i.e. retention after HG) to retention after Noise (*p*=0.022). However, a closer look into the results revealed interesting differences in learning based on subjects’ initial skill level. The participants were divided into two groups according to the mean tracking error during retention after the HG mode. Non-skilled subjects created significantly larger errors during retention after HG than skilled subjects (Figure 4, *p*<0.001). Non-skilled subjects significantly reduced the errors after training with NG (*p*=0.028), and Ea (*p*=0.020), and showed a tendency in learning after Noise (*p*=0.099). Although the error reduction after Ea seemed greater than after the other active modes (Figure 4), the differences were not significant (Ea vs. Noise: *p*=0.290, Ea vs. NG: *p*=0.409). Initially skilled subjects did not learn with any training strategy. They only reduced somehow the error after training with Noise, to a level significantly smaller than the non-skilled subjects.

The performance adaptation during training also presented differences depending on subjects’ initial skill level. The comparison of the tracking error during the first and last thirds of each training mode showed a tendency of error reduction when training with Ea (*p*=0.088), while no differences were found in the other modes. However, when looking into adaptation in the initially less skilled subjects, a significant error reduction while training with Ea was found (*p*=0.044), while a significant increase of the error during Noise was found (*p*=0.019). No differences were found during adaptation in the skilled group.

### Aftereffects during retention

**Figure 5:**
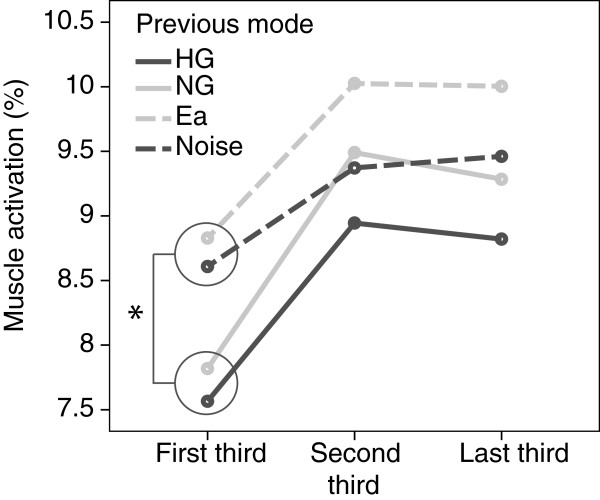
**Muscle activation of the non-dominant leg during all thirds of retention after different training modes: Haptic guidance, No guidance, Error amplification and Noise disturbance.** * *p*<0.05.

We compared the muscle activation during retention after the two challenge-based modes (i.e. Ea and Noise) to the two conditions without any disturbance (i.e. HG and NG). Although subjects were informed not to expect any error disturbance during retention, the muscle activation of the active leg was higher after the Ea and Noise modes (*p*=0.038). A closer look into the muscle activation during retention revealed that the differences between the groups with and without error perturbations appeared mostly during the first third of the retention movements (Figure 5, *p*=0.011). The difference between groups faded towards the end of the last third of the retention session, when the difference, although still existent, was non-significant (*p*=0.075). Interestingly, the muscle activation of the passive leg was during the first third significantly lower after the challenge-based modes (*p*=0.024). Similarly, the difference faded as movement progressed, until no significant differences were appreciated during the last third.

The aftereffects also showed differences depending on subjects’ initial skill level. Both groups showed more muscle activation in the active leg just after the disturbance conditions (first third of retention), however only for those initially less skilled the difference was significant (*p*=0.005). The muscle activation of the passive leg was during the first third of the retention almost significantly lower after the conditions with disturbance, but only in the less-skilled subjects (*p*=0.056). Differences in both legs faded as movement progressed, until no significant differences were observed in the last third.

## Discussion

We designed and implemented four different training strategies for an fMRI compatible robotic device that can assist or resist during stepping: i) haptic guidance, ii) no guidance, iii) error amplification, and iv) noise force disturbance. In haptic-guidance mode, MARCOS guided the gait pattern, while the subject remained passive. In no-guidance mode, the subject was in charge of the movement generation. In error-amplification mode, the robot amplified the tracking error created by the subject, adding a force on the knee proportional to the tracking error. In noise-force-disturbance mode, a random force was added to the no-guidance mode in order to disturb the subject’s movement.

As hypothesized, the error-amplification mode was the most difficult training strategy, as suggested by the highest muscular effort during training. Furthermore, the tracking error in Ea was significantly higher than in all other modes. However, and contrary to expected, we did not find significant differences in the mean tracking error between Noise and NG. The noise disturbance had the effect of a short and fast change in the movement’s smoothness. Thus, perhaps due to the short period of time that the force was applied the overall tracking error did not increase significantly. There was, however, a tendency of higher muscle activation in Noise compared to NG, suggesting that although the random noise disturbance was not high enough to influence the mean absolute error, it was at least more physically demanding. In fact, the higher muscular effort visible in the Noise mode compared to the NG mode may explain why the tracking error during the Noise mode was similar to NG: subjects were able to cancel the tracking errors using muscular effort.

Subjects adapted to the error-amplification mode during training, as suggested by the reduction of tracking error from the first to the last third of the training session. Adaptation to error amplification was expected, since previous research on motor learning suggested the formation of an internal model when training under error-amplification strategies [13]. Subjects did not adapt to Noise disturbance. This finding was also expected, since the magnitude of the disturbing force was random and an anticipatory model formation was not possible. We found, however, that the tracking error increased during training, at least in the initially non-skilled subjects. This performance degradation may be explained by the reduced compensation of the random forces that destabilized the movement due to muscle fatigue.

Research in motor learning has stated that increasing the task difficulty during training can be beneficial for motor learning of specially simple tasks [33]. Thus, we hypothesized that training with challenge-based strategies would result in better motor learning. Interestingly, subjects seemed to learn only when trained with the Noise mode, even if the Noise did not increase the mean tracking error during training. This is in line with a recent study that found that randomly-varying feedforward forces that disturb subjects’ movements resulted in better tracking than unassisted training and training with error-amplification. A possible rationale for the positive effect of noise in motor learning is that subjects could not anticipate the disturbing force, and thus they remained concentrated during training, even if the locomotion task was quite simple. In fact, the constantly greater muscle activation during training with noise supports this rationale. However, subjects learned how to adapt to the error amplification, and thus the task may have required less concentration, hampering motor learning.

Motor learning depended on the subjects’ initial skills. This finding is in line with recent studies [19,34,35]. Initially non-skilled subjects learned to perform the task after training with error amplification and without any error augmented strategy, and showed a tendency of better learning with noise force disturbance. Initially more skilled subjects only reduced the tracking error after training with Noise, although not significantly. A possible rationale why error amplification seemed to produce the best learning in the less skilled subjects might be due to the logical behavior of the controller. In contrast to the noise force disturbance mode, the perturbations of the error amplification mode depended on the subjects’ performance: i.e. only existing errors were amplified, with higher amplification for larger errors. Thereby, contrary to the other active modes, subjects could detect their errors and correct them. However, the error reduction after training with Noise was not significant. Thus, Noise seemed to limit learning in initially non-skilled subjects, perhaps because it made the task too demanding and frustrating, and thereby increased fatigue and reduced motor learning. Skilled subjects may not have been challenged enough during error amplification, since they were systematically making less errors. However, as the Noise mode was independent of the subjects’ performance, it increased subjects awareness and attention when performing the locomotion task.

Therefore, with this experiment we confirmed our hypothesis that error-amplification and noise-disturbance modes improve motor learning of this especially simple task. We found, however, that care has to be taken when choosing the challenge-based mode depending on the subject’s initial skill level. Ea seemed to be more suitable to train non-skilled subjects, while Noise seemed to improve learning in more skilled subjects.

Subjects remained passive during the HG mode, as can be assessed from the significantly lower muscle activation and small mean position error compared to the active modes. However, although subjects were requested to remain their dominant leg passive through the experiment, they found it challenging to relax the leg when errors were amplified or randomly added to the active leg. In fact, subjects appeared to actively try to synchronize the passive leg with respect to the active leg (instead of the active with respect to the passive) when some kind of disturbance was applied to the active knee. This might be an important finding for the interpretation of brain activation results in further experiments. Active effort of the supposedly passive leg may result in bilateral activation of the sensorimotor cortex, even if the task to be performed requires only activation of the non-dominant leg [26].

Although subjects were informed to do not expect any disturbances during retention after training, a short term aftereffect was found after the challenge-based modes. Subjects were apparently more alert after the challenge-based conditions, and therefore had a higher muscular activation in the active leg. Interestingly, in the dominant (passive) leg the muscle activation was significantly lower during the first third of retention after the conditions with disturbance. Probably, the difficulty to remain passive was higher during these more challenging conditions, and thus when the error disturbances were removed, subjects already have learned how to keep the dominant leg passive.

The experimental design suffers from some limitations. First, comparison of muscle activation with other studies is limited because the EMG data was normalized to the maximum of the corresponding muscle measured during the entire experiment, rather than the maximum voluntary contraction of each muscle. Secondly, in order to better assess the aftereffects once the error perturbations are removed, subjects should not have been informed about the absence of perturbations during retention. The aftereffects may have been reduced due to subject’s prior knowledge of the retention movement. Subjects were probably mentally relaxed when they started the sustained movement and, therefore, were less alert than they would have been without any a priori information.

Several studies have suggested that motor adaptation in healthy subjects has similarities to motor learning in patients [23,36]. If this is applicable to the strategies investigated in this study with MARCOS can nevertheless not be assured. Further studies with severely affected neurologically patients need to be performed. The impacts of different training strategies on motor learning may differ in neurological patients. They probably have a lower initial skill level and therefore, their optimal challenge point (i.e. where subjects show the best motor learning) may be at a different level [33]. Hypothetically, the haptic guidance mode may be especially suitable for more disabled patients, as suggested in [19,34,35].

## Conclusions

Adding random force disturbances during training appeared to increase attention, and therefore improve motor learning. Error amplification seems to be the most suitable strategy for initially less skilled subjects, perhaps because subjects could detect their errors and correct them. Strategies that reduce or do not amplify errors reduce muscle activation during training and limit motor learning. Error strategies have a great potential to evoke higher muscle activation and provoke better learning of especially simple motor tasks.

It is still an open question how different rehabilitation strategies contribute to restorative processes of the central nervous system. Evaluation of brain regions involved in learning can provide valuable information on the observed behavioral outcomes related to learning processes. The results from studying the particular brain regions involved in learning might have important therapeutic implications in terms of tailoring motor training conditions to the anatomical location of a focal brain insult. To achieve this goal, we aim to evaluate the brain regions involved in learning when training with the different forms of robotic guidance and error amplification applying fMRI.

## Competing interests

The authors declare that this work was done in absence of competing interests.

## Authors’ contributions

LMC, LJ and RR contributed to the experimental design and project supervision. LMC and JS participated in the study design, data acquisition and data analysis. LMC and JS prepared the manuscript. All authors read and approved the final manuscript.
